# A case of acquired thrombotic thrombocytopenic purpura following near-drowning 

**DOI:** 10.5414/CNCS111301

**Published:** 2024-05-03

**Authors:** Natnicha Leelaviwat, Shanti Parkash, Sharma Prabhakar

**Affiliations:** 1 Department of Internal Medicine, and; 2 Division of Nephrology and Hypertension, Texas Tech University Health Sciences Center, Lubbock, TX, USA

**Keywords:** thrombotic thrombocytopenic purpura, near-drowning, refractory thrombotic thrombocytopenic purpura

## Abstract

A 19-year-old woman with a history of asthma presented with acute confusion following a near-drowning event 2 weeks prior to admission. She was found to have severe thrombocytopenia and microangiopathic hemolytic anemia (MAHA). The treatment for thrombotic thrombocytopenic purpura (TTP) was started on the day of admission due to high clinical suspicion. Subsequent workup confirmed a diagnosis of TTP with no clear etiology except the near-drowning incident. TTP following a near-drowning event has never been reported in the literature. Furthermore, she developed refractory TTP that required reinitiation of therapeutic plasma exchange and rituximab. After discharge, the patient had been doing well over a year of follow-up without remission.

## Introduction 

Thrombotic thrombocytopenic purpura (TTP) is one of the thrombotic microangiopathy (TMA) syndromes that result from severe ADAMTS13 deficiency leading to microangiopathic hemolytic anemia (MAHA) and severe thrombocytopenia. The etiology can be either inherited or acquired [1, 2]. TTP is associated with several pathophysiologic conditions such as pregnancy, autoimmune disorders, and malignancy [1, 3]. We present here a patient who developed TTP without any of such predisposing conditions. Interestingly, the syndrome followed a near-drowning event. Unusual and unreported association between drowning and TTP and the potential mechanisms contributing to such association are the focus of this manuscript. 

## Case description 

A 19-year-old female with a history of asthma was brought to the emergency department due to confusion for 5 hours. The patient had experienced a near-drowning event in a small rural town for almost 15 minutes 2 weeks prior to this admission. Initially, she was evaluated at the emergency department (2 weeks ago) with blood work and chest X-ray which were normal, and was discharged home. However, 3 days following the drowning event, she began to experience headaches, nausea, and fatigue. She also noticed dark-colored urine, and jaundice started 6 days after the drowning event. Her symptoms continued until the day of admission. Five hours prior to this admission, she started developing confusion and agitation. She had no family history of hematologic diseases. Upon examination on the day of admission, she was afebrile with a blood pressure of 140/127 mmHg and a heart rate of 102 per minute. Her oxygen saturation was 97% on room air. Generalized petechiae were present, but no nuchal rigidity was observed. Initial laboratory tests (Table 1) were significant for leukocytosis (WBC 26 K/mcL), severe thrombocytopenia (8 K/mcL), and hemolytic anemia with MAHA. The patient was intubated for airway protection and was admitted to the intensive care unit. The differential diagnosis included TTP, hemolytic uremic syndrome, intracranial infection, and disseminated intravascular coagulation. She was started on broad-spectrum intravenous antimicrobial therapy with vancomycin and meropenem to cover possible central nervous system infection. The dose of vancomycin and meropenem were adjusted for kidney function, and vancomycin level was monitored. Both vancomycin and meropenem had been continued for a total of 14 days. 

**Figure 1.**
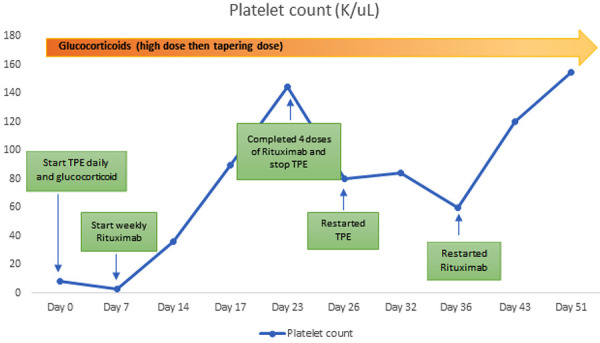
Platelet count over the course of treatment.

The patient was also initiated on therapeutic plasma exchange (TPE) and high-dose glucocorticoids on the day of admission. Her mental status improved the following day. However, on the 3^rd^ day of hospitalization, the patient developed acute kidney injury (AKI). Creatinine increased from 0.8 to 1.4 mg/dL. Fractional excretion of sodium was 3.2% with a urine sodium level of 183 mM/L, consistent with acute tubular necrosis (Table 1). Treatment for TTP was continued, while potential infections were covered by broad-spectrum antibiotics, and nephrotoxic agents were avoided. Within 4 days, creatinine returned to baseline. All tests for infectious (Table 2) and autoimmune etiologies (Table 1) came back negative. CT scans of the head, chest, abdomen, and pelvis showed no abnormalities. ADAMTS13 level was reported to be < 1%, along with the presence of antibodies against ADAMTS13 after 6 days of admission, confirming the diagnosis of TTP. In addition to plasmapheresis and glucocorticoids, the patient was also started on weekly rituximab 6 days after the initiation of therapy, in view of insignificant response. Platelet count began to improve after receiving a 2^nd^ dose of rituximab and increased to above 150,000/µL (Figure 1) following the completion of the 4^th^ dose of rituximab. Consequently, rituximab and TPE were discontinued. However, after the discontinuation of rituximab and TPE, 3 days later, the patient manifested the picture of TTP again indicating a refractory disease resulting in the reinitiation of TPE. Rituximab was reinitiated 10 days after TPE was restarted due to unresponsiveness (Figure 1). There was a notable response after the 1^st^ dose of rituximab re-initiation, particularly in the platelet count before the decision was made to start caplacizumab. Caplacizumab is a humanized anti-von Willebrand Factor (vWF) Nanobody which is indicated for the treatment of acquired TTP. 

She was discharged home on a tapering dose of oral prednisone on day 51 of her admission, with a platelet count of 155,000 and creatinine levels close to her baseline. Oral prednisone was discontinued after a month of discharge. She continues to do well without signs of remission after a year of follow-up. 

## Discussion 

TTP is a life-threatening blood disorder that results in the formation of microthrombi throughout the body. These microthrombi lead to decreased blood flow to various organs and cause multi-organ damage. TTP is manifested by the pentad of microangiopathic hemolytic anemia, thrombocytopenia, AKI, fever, and neurological issues. Signs and symptoms include petechiae, purpura, jaundice, nausea and vomiting, headache, altered mental status, and coma if the brain is involved. TTP can be triggered by conditions in which vWF levels are increased such as pregnancy, inflammatory disorders, autoimmune disorders, and cancers. The vWF is a glycoprotein that has a very important role in primary hemostasis [4]. The ultra-large active vWF binds to platelets which in turn leads to the formation of platelet microthrombi throughout the body to the point where it consumes the platelets and causes thrombocytopenia. ADAMTS13 protein is a metalloproteinase and is involved in enzymatic cleavage of ultra-large active vWF multimers into vWF monomers which have less thrombogenic activity [5]. 

There are two types of TTP, inherited or congenital TTP (cTTP) and acquired or immune-mediated TTP (iTTP). cTTP is a very rare autosomal recessive disorder caused by an inherited mutation in the gene encoding ADAMTS13 protein at chromosome 9q34, whereas iTTP is caused by the formation of autoantibodies against ADAMTS protein. 

TTP is diagnosed by clinical symptoms in combination with abnormal laboratory findings of low platelet count along with increased bleeding time, abnormal laboratory workup of hemolysis such as low haptoglobin, elevated lactate dehydrogenase LDH, normocytic anemia, and schistocytes on peripheral smear. ADAMTS13 activity is usually < 10% of normal. TTP is considered a medical emergency due to its potential to rapidly escalate into a life-threatening condition. The formation of widespread blood clots, particularly in small blood vessels, can lead to severe organ damage and failure. Urgent intervention is required to prevent serious complications such as stroke, kidney failure, and heart damage. Without prompt treatment, TTP can be fatal [6]. 

The intriguing association between acquired TTP and near-drowning in this case presents a unique and rare clinical scenario that challenges our understanding of the complex interplay between hemostasis, inflammation, and ischemic injury. While the exact pathophysiological mechanisms remain unclear, several possible hypotheses can be postulated based on the available literature. 

Severe hypoxia and systemic inflammatory response: Near-drowning events are characterized by severe hypoxia, which can result in multi-organ dysfunction due to oxygen deprivation. Hypoxia triggers a cascade of events, including the release of pro-inflammatory cytokines and activation of immune responses. This systemic inflammatory response could play a role in endothelial dysfunction, a hallmark of TTP pathogenesis. The endothelium, when subjected to inflammation, expresses adhesion molecules and pro-coagulant factors that contribute to platelet activation and aggregation. The ischemic insult from near-drowning might exacerbate this inflammatory response, fostering an environment conducive to microvascular thrombosis [7]. 

Endothelial damage and platelet aggregation: The severe hypoxia and reoxygenation during resuscitation efforts in near-drowning cases can inflict significant damage to the endothelial lining of blood vessels. The resulting endothelial injury exposes subendothelial structures, leading to platelet adhesion and activation. Additionally, the release of vWF from the endothelium, combined with its impaired cleavage due to reduced ADAMTS13 activity, can promote the formation of unusually large vWF multimers. These multimers, known to initiate platelet aggregation and thrombus formation, contribute to the microangiopathic hemolytic anemia characteristic of TTP [8]. 

Hypothermia and coagulation cascade activation: Hypothermia is a common consequence of submersion in cold water during near-drowning events. Cold exposure can alter coagulation dynamics, leading to enhanced platelet aggregation and activation of the coagulation cascade. Cold-induced vasoconstriction and reduced blood flow contribute to stasis, increasing the likelihood of thrombus formation. Furthermore, hypothermia can impair the function of enzymes involved in coagulation regulation, potentially leading to a pro-thrombotic state. The interplay between hypothermia, impaired enzyme function, and endothelial damage could synergistically contribute to microvascular thrombosis in TTP [9]. 

Anti-ADAMTS autoantibodies: Autoantibodies against ADAMTS13 are necessary for the development of iTTP [10]. While risk factors for the development of autoantibodies are not clearly defined, certain factors such as female sex and African ancestry are known to predispose individuals to these antibodies. Secondary iTTP can be associated with various infections (HIV, hepatitis C, *Helicobacter pylori*, influenza A), autoimmune conditions, and acute stressors [11]. Acute stressors or acute inflammatory response could increase the risk of ADAMTS deficiency by promoting the release of ultra-large VWF multimers from endothelial cells and inhibiting their cleavage by ADAMTS13, as demonstrated in in vitro studies [12]. Although the relationship between drowning and the development of anti-ADAMTS13 antibodies has never been reported, besides the above-mentioned hypothesis, drowning might also trigger an acute episode of TTP through the acute inflammatory response mechanism. Furthermore, the patient’s female sex may predispose to the development of anti-ADAMTS13 autoantibodies. 

Drug-induced thrombotic microangiopathy (DITMA): Cocaine use has been reported to be associated with the clinical syndrome of MAHA, often referred to as DITMA, which can clinically mimic TTP. However, ADAMST13 activity is usually normal in DITMA, and there is no evidence of anti-ADAMTS13 antibodies [13, 14]. Our patient tested positive for cannabinoids in a urine drug screen. However, the ADAMTS13 level was severely decreased, along with the presence of antibodies against ADAMTS13. Moreover, the occurrence of TTP manifestation after the discontinuation of TTP treatment made the diagnosis of DITMA less likely. 

TPE remains the cornerstone of acquired TTP management, as it aims to remove pathogenic autoantibodies, replenish ADAMTS13 levels, and improve microcirculation. Additionally, the administration of glucocorticoids helps to suppress immune-mediated destruction of ADAMTS13. In cases of acquired TTP following near-drowning, early initiation of TPE and glucocorticoid therapy may be crucial to prevent the progression of microvascular thrombosis and organ damage [15]. The treatment of refractory TTP poses a significant challenge. In cases resistant to standard therapies like plasma exchange and glucocorticoids, more aggressive approaches are often required. Rituximab, an anti-CD20 monoclonal antibody, targets B cells involved in autoantibody production, aiming to halt the autoimmune process. Additionally, caplacizumab, an anti-vWF nanobody, inhibits platelet aggregation and microthrombus formation. Emerging therapeutic options include recombinant ADAMTS13 and immune modulators [16]. 

## Conclusion 

TTP is frequently associated with autoimmune disorders, cancers, infections, inflammatory conditions, and pregnancy. Absence of these conditions before the drowning event in our patient and the fact that it all started after the patient had a near-drowning experience make this case a unique reportable case since it has never been reported to the best of our knowledge. Furthermore, our patient developed refractory TTP after discontinuation of rituximab and TPE which is a rare occurrence. 

## Authors’ contributions 

Case description and review literature: N.L.; Review literature and case discussion: S.P.; Supervision and revision: S.P. N.L. and S.P. take responsibility that this study has been reported honestly, accurately and transparently, and accepts accountability for the overall work by ensuring that questions pertaining to the accuracy or integrity of any portion of the work are appropriately investigated and resolved. 

## Funding 

The author(s) received no financial support for the research, authorship, and/or publication of this article. 

## Conflict of interest 

We have no conflict of interest to disclose. 

**Table 1.** Laboratory test results.

**Investigation**	**Laboratory test results**	**Reference range**
White blood cell (K/µL)	26.34	3.98 – 10.04
Hemoglobin (g/dL)	7.2	11.2 – 15.7
Platelet (K/µL)	8	182 – 369
Iron panel		
Iron level (mcg/dL)	303	37 – 145
Iron-binding capacity (mcg/dL)	351	250 – 450
Ferritin level	1321	13 – 150
Transferrin saturation (%)	86	5 – 62
Transferrin (mg/dL)	226	200 – 360
Folate (ng/dL)	12.9	4.6 – > 20
Vitamin B 12 (pg/dL)	549	232 – 1245
Prothrombin time (s)	15.2	9.4 – 12.5
Partial thromboplastin time (s)	25.4	26 – 36.5
INR	1.33	> 5.0
D-dimer (ng/mL)	4,504	≤ 500
Fibrinogen (mg/dL)	191	200 – 393
LDH (U/L)	2,690	135 – 225
Haptoglobin (mg/dL)	< 8	43 – 212
Reticulocyte (%)	12.35	0.5 – 1.8
Direct Coombs	Negative	Negative
ADAMTS13 activity (%)	< 1	> 60
ADAMTS13 inhibitor (BEU)	4.0	< 0.4
Serum sodium (mmol/L)	140	136 – 145
Serum potassium (mmol/L)	3.8	3.5 – 5.1
Serum calcium (mg/dL)	8.9	8.8 – 10.5
Serum magnesium (mg/dL)	1.8	1.6 – 2.4
Serum phosphorus (mg/dL)	3.2	2.7 – 4.5
Serum creatinine (mg/dL)	1.0	0.5 – 1.2
Blood urea nitrogen (mg/dL)	25	6-20
Aspartate transaminase (IU/L)	83	5 – 37
Alanine transaminase (IU/L)	25	5 – 41
Alkaline phosphatase (IU/L)	87	35 – 129
Total bilirubin (mg/dL)	5.4	0 – 1.0
Direct bilirubin (mg/dL)	0.4	0 – 0.2
Ammonia level (mcmol/L)	11	11 – 51
TSH (mIU/L)	0.79	0.7 – 4.2
Serum Beta-HCG	Negative	–
Antinuclear antibody	Negative	Negative
Antiphospholipid antibody testing (lupus anticoagulant, anticardiolipin antibodies, anti-β-glycoprotien I antibodies)	Negative	Negative
Rheumatoid factor	Negative	Negative
Peripheral blood smear	Normocytic/normochromic anemia Moderate schistocytes Leukoerythroblastosis Neutrophilia Monocytosis Thrombocytopenia	–
Acetaminophen level (mcg/mL)	< 5.0	10 – 30
Salicylate level (mg/mL)	< 0.3	0 – 20
Urine drug screen	Cannabinoid positive	Negative
Urinalysis (day 3)	Red color, WBC 16/HPF, RBC 18/HPF, large blood, protein 100 mg/dL	–
Fractional excretion of sodium (day 3) (%)	3.2	–
Urine sodium (day 3) (mmol/L)	183	28 – 287

**Table 2.** Infectious workup and serology.

**Infectious workup**	**Results**
Blood culture	No growth at day 5
Urine culture	Normal urogenital flora
Respiratory viral panel	Not detected
Stool enteric panel	Not detected
Stool Shiga toxin	Not detected
Stool culture	No growth
Cryptococcal antigen test	Negative
Coccidioides	Negative
Urine *Histoplasma* galactomannan antigen (ng/dL)	< 0.2
HIV	Non-reactive
Viral hepatitis profile	
Hepatitis A Ab IgG	Reactive
Hepatitis A Ab IgM	Non-reactive
Hepatitis B core Ab IgG	Non-reactive
Hepatitis B core surface Ab	Negative
Hepatitis B core surface Ag	Non-reactive
Hepatitis C Ab IgG	Non-reactive
